# Roles of endophytic fungi in plant resilience under abiotic stress: A mechanistic review with implications for climate-smart agriculture

**DOI:** 10.1080/15592324.2025.2578712

**Published:** 2025-11-04

**Authors:** Ndivhuwo Ramatsitsi, Alen Manyevere

**Affiliations:** Department of Agronomy, University of Fort Hare, Alice, South Africa

**Keywords:** Antioxidant defence, plant abiotic stressors, plant-fungi interactions, stress tolerance, sustainable crop production

## Abstract

Endophytic fungi have emerged as vital allies in enhancing plant resilience to abiotic stresses, offering significant potential for climate-smart agriculture (CSA). This mechanistic review synthesises physiological, biochemical, and molecular pathways through which these symbionts mitigate stress, emphasising mechanistic understanding over descriptive diversity. Key mechanisms include osmotic regulation, ion homeostasis, antioxidant defence, hormonal modulation, and epigenetic reprogramming. As demonstrated by studies on *Trichoderma harzianum*, *Fusarium solani*, and *Piriformospora indica*, such interactions allow plants to sustain photosynthesis, nutrient uptake, and growth when subjected to drought, salinity, heat, and heavy metal stress. Comparative insights highlighted lineage-specific strategies: Ascomycota display broad-spectrum regulation through metabolite production and hormonal control; Basidiomycota specialise in root–fungus signalling and resource acquisition; and Zygomycota contribute primarily to nutrient mobilisation and rapid colonisation. Collectively, these insights reveal that endophytes act as “hidden regulators” of plant stress resilience. Integration of fungal symbionts into CSA practices holds considerable potential for improving productivity, adaptation, and mitigation, particularly in stress-prone agroecosystems. Yet, as several sources have argued, key gaps remain including field performance is inconsistent across host genotypes, beneficial and pathogenic traits sometimes overlap, and inoculant mass production is still limited. Addressing these challenges will necessitate omics-driven innovations, efficient delivery methods, improved relationships between empirical biological research and on-farm application, and policy frameworks. Overall, this synthesis highlights endophytic fungi as essential partners in building resilient and sustainable food systems under changing climates.

## 1. Introduction

Abiotic stresses including drought, salinity, heat, and heavy metal toxicity are among the most significant constraints on global agricultural productivity. These environmental pressures disrupt physiological processes, such as photosynthesis, nutrient uptake, and water relations, ultimately reducing crop yield and quality.^1^ For instance, salinity stress disrupts cellular ion balance and water uptake, while drought accelerates oxidative stress and limits carbon assimilation.^2^ The impacts of such stressors are being intensified by climate change, which is projected to increase their frequency and severity in both temperate and tropical agroecosystems.^3^ Abiotic stress is a major driver of food insecurity in sub-Saharan Africa and other marginalised regions, primarily through their impact on yield stability of staple crops such as maize (*Zea mays*), rice (*Oryza sativa*), and wheat (*Triticum aestivum*). Conventional methods, such as chemical inputs, breeding, and irrigation, offer some respite although they may fall short when presented with limited resources or shifting temperatures.^4^ Thus, there is a pressing need for creative strategies that promote sustainability and increase stress resistance.

Climate-smart agriculture (CSA) is a holistic framework developed by the Food and Agriculture Organisation (FAO) to achieve three interconnected goals: (i) sustainably increase agricultural productivity and incomes, (ii) enhance adaptation and resilience to climate variability, and (iii) reduce or remove greenhouse gas emissions where possible.^5^ These pillars; productivity, adaptation, and mitigation, serve as benchmarks for evaluating the climate relevance of agricultural innovations. CSA emphasises the integration of ecological processes, including soil microbial dynamics, into farming systems to improve adaptation to abiotic stress Within CSA, ecological processes, such as soil microbial dynamics, are central to improving adaptation to abiotic stress.^6^ Agroecosystem resilience is often promoted through practices such as crop diversification, conservation tillage, and organic amendments. Yet, microbial symbionts, most notably endophytic fungi, remain underexplored in the context of CSA.^7^ Endophytic fungi are microorganisms that colonise internal plant tissues without causing disease, often conferring mutualistic benefits to their hosts.^8^ Incorporating fungal endophytes into CSA can provide “biological insurance” against climate variability, reducing reliance on costly agrochemicals while improving soil health and crop vigour. Moreover, beyond immediate stress mitigation, fungal endophytes align with CSA principles by supporting long-term ecosystem functions such as nutrient cycling and carbon sequestration.^9^ Recognising endophytes as part of CSA’s biological toolkit not only strengthens adaptive capacity but also contributes to sustainable intensification, particularly in smallholder farming systems that are most vulnerable to climate shocks.

Unlike mycorrhizal fungi, which have been extensively studied in stress adaptation,^10^^,^^11^ endophytes remain relatively underexplored despite their widespread occurrence across ecosystems. These fungi have been shown to enhance host tolerance to drought, salinity, heat, and heavy metal stresses by modulating physiological, biochemical, and molecular processes. For example, certain *Trichoderma* species, such as *T. harzianum*, improve drought resilience in barley (*Hordeum vulgare*) through antioxidant enzyme activation,^12^ while *Piriformospora indica* enhances nutrient uptake and root growth of date palm (*Phoenix dactylifera*) under salinity stress.^13^ Importantly, endophytes display high functional diversity, with distinct mechanisms even among closely related taxa. Their dual role in both direct plant protection and indirect ecosystem functions makes them promising candidates for integration into sustainable agriculture.^14^^,^^15^ However, a mechanistic understanding of how these fungi operate under abiotic stress is still developing and remains fragmented across studies.

Previous studies on endophytes have largely focused on cataloguing plant–fungus associations or discussing broad agricultural applications.^9^^,^^14^ While these contributions are valuable, there is a pressing need to synthesise findings through a mechanistic lens. A mechanistic review goes beyond listing case studies to unpack the biochemical, physiological, and molecular pathways through which endophytic fungi regulate stress responses in plants. In this regard, this review addresses key questions: (i) What physiological, biochemical, and molecular mechanisms do endophytic fungi use to enhance plant resilience under abiotic stressors? (ii) How do stress-mitigation strategies differ among Ascomycota, Basidiomycota, and Zygomycota, and what lineage-specific traits underpin these differences? (iii) Which plant traits are consistently modulated by endophytes across stress types? and (iv) How can mechanistic insights into endophytic fungi be translated into CSA practices to improve productivity, adaptation, and mitigation? The review focuses on drought, salinity (osmotic), heat, and heavy-metal stress, major global constraints to crop productivity because they correspond to water deficit, ionic imbalance, thermal injury, and redox stress that align with CSA’s three pillars. By mechanistically examining pathways such as antioxidant defence, ion homoeostasis, osmotic balance, hormonal regulation, and epigenetic modifications, this review aims to clarify how different fungal lineages contribute to plant resilience. Special attention is given to the comparative roles of Ascomycota, Basidiomycota, and Zygomycota, highlighting lineage-specific strategies in stress mitigation. This mechanistic synthesis will provide a foundation for leveraging fungal endophytes as practical allies in climate-smart and sustainable agriculture.

## 2. Mechanistic basis of endophytic fungal mediation in abiotic stress

### 2.1. Osmotic balance and water relations

Water scarcity is one of the most severe abiotic stresses observed in crop production, and endophytic fungi can significantly enhance plant drought resilience by modulating osmotic balance and water relations. Several endophytes induce the accumulation of compatible solutes such as proline, trehalose, and soluble sugars that help maintain cell turgor during dehydration.^16^ Javed et al.^17^ observed that inoculating *Moringa oleifera* L. with endophytic *Microdochium majus*, *Meyerozyma guilliermondi*, and *Aspergillus aculeatus* exhibited a significant improvement in growth attributes under drought stress. This improved growth was attributed to increase in primary and secondary metabolites including proteins, sugars, lipids, phenols, flavonoids, proline, indole acetic acid (IAA), gibberellic acid (GA), salicylic acid (SA), and ascorbic acid. In yet another study, Sorahinobar et al.^12^ reported that priming *H. vulgari* seeds with *T. harzianum* through increased superoxide dismutase, peroxidase, polyphenol oxidase activity, total phenolics, flavonoids, and anthocyanins; thereby improving water uptake efficiency under limited water availability. Fungal metabolites also stimulate root architectural changes, such as increased root branching and deeper root penetration, which improve soil water extraction.^18^ In addition, some endophytes such as *Salicaceae* [unspecified species] have been shown to reduce stomatal conductance under water deficit in black cottonwood (*Populus trichocarpa*), striking a balance between water conservation and CO₂ assimilation.^19^ Together, these mechanisms allow host plants to maintain photosynthetic activity and growth under drought stress. Such fungal-mediated osmotic regulation aligns with CSA strategies, particularly in arid and semi-arid agroecosystems where water scarcity is a dominant production constraint.

### 2.2. Ion homoeostasis and salt stres

Salinity stress disrupts cellular ion balance by causing toxic accumulation of sodium (Na⁺) and chloride (Cl⁻) ions, which interfere with potassium (K⁺) uptake and essential metabolic processes.^20^ Endophytic fungi play a critical role in regulating ion homoeostasis to mitigate salt stress. For example, *Talaromyces adpressus, T. argentinensis* and *A. welwitschiae* endophytes activated Na⁺/K⁺ transporters in *O. sativa* subjected to salinity, enabling plants to maintain favourable ionic ratios even in saline soils.^21^ Likewise, *P*. *indica* promoted sequestration of excess sodium into vacuoles, preventing cytoplasmic toxicity in thale cress (*Arabidopsis thaliana*).^22^ Additionally, fungal metabolites such as organic acids chelate ions, reducing their bioavailability in plant tissues. Endophytes also enhance selective ion uptake, ensuring that beneficial nutrients like potassium and calcium are maintained under salt stress conditions.^23^ These mechanisms not only support plant growth in saline-prone regions but also reduce the need for chemical soil amendments. Integrating salt-tolerant endophytic fungi into CSA frameworks holds promise for enhancing productivity in coastal and irrigated farmlands increasingly threatened by salinisation. Although promising, the application dynamics and persistence of endophytic fungi in soil ecosystems remain poorly understood. Few studies have quantified and reported on the density of inoculated propagules or metabolites that are transferred from internal plant tissues into the rhizosphere or bulk soil after colonisation or senescence.^24^ Field survival is often constrained by competition with native microbes, environmental variability, and limited re-colonisation efficiency.^25^^,^^26^ These factors may lead to inconsistent performance across seasons or sites, underscoring the need for further work on formulation stability and ecological risk assessment before large-scale application.

### 2.3. Antioxidant defence systems

Abiotic stresses such as drought, salinity, and temperature extremes commonly lead to the overproduction of reactive oxygen species (ROS), including superoxide radicals, hydrogen peroxide, and hydroxyl ions. Excess ROS cause oxidative damage to lipids, proteins, and nucleic acids, impairing plant growth and survival.^1^ Endophytic fungi mitigate oxidative damage by activating and enhancing host antioxidant defence systems. In thyme (*Thymus vulgaris*) inoculated with *F. solani*, *Cladosporium puyae*, and *Curvularia australiensis* upregulate enzymes such as superoxide dismutase (SOD), catalase (CAT), and ascorbate peroxidase (APX), which detoxify ROS.^27^ This activation is mediated through fungal elicitors that trigger host redox signalling and hormonal pathways, particularly those involving SA, jasmonic acid, and nitric oxide, leading to the transcriptional activation of antioxidant-related genes (SOD, CAT, and APX).^27^ For instance, *T. harzianum* secretes low-molecular-weight peptides and volatile organic compounds that act as ROS sensors, initiating MAP-kinase cascades that induce SOD and CAT synthesis in barley under drought stress.^12^ Likewise, colonisation by *P. indica* in *A. thaliana* stimulates SA and nitric oxide accumulation, enhancing APX and glutathione reductase activities.^28^ Some endophytes also produce their own antioxidant metabolites, including phenolics, flavonoids, and glutathione, which provide additional protective capacity.^22^ For example, endophytic *Fusarium solani* SD5. has been shown to increase glutathione reductase activity in stressed plants, maintaining redox balance.^29^ By strengthening the antioxidant network, endophytes enable plants to withstand prolonged stress exposure without succumbing to cellular damage. This mechanism is particularly relevant under climate variability, where oxidative stress episodes occur in parallel with drought and heat waves.

### 2.4. Hormonal modulation

Plant hormones are central regulators of stress perception and adaptation, and endophytic fungi frequently manipulate hormonal signalling to enhance resilience. One common mechanism is the modulation of abscisic acid (ABA), the key stress hormone involved in stomatal closure, osmotic adjustment, and drought response.^19^ Fungal endophytes such as *A. japonicu* and *A. flavus* have been demonstrated to either stimulate ABA biosynthesis in host tissues and/or produce ABA themselves, fine-tuning water stress responses in soybean (*Glycine max*) and sunflower (*Helianthus annuus*).^30^^,^^31^ An auxin producing endophytic fungi, *Cyanodermella asteris*, was reported to synthesise IAA in *A. thaliana*,^32^ which promote root elongation and lateral branching, improving water and nutrient acquisition under stress.^33^ Cytokinin and GA production by endophytes can counteract stress-induced growth inhibition, maintaining biomass accumulation during adverse conditions. Another important aspect is ethylene regulation; for instance, Rauf et al.^34^ established production of 1-aminocyclopropane−1-carboxylate deaminase by *T. asperellum* in *T. aestivum*, lowering ethylene levels and preventing premature senescence under water logging stress. Through these hormonal interactions, endophytes act as “hidden regulators” of plant development, coordinating responses that allow crops to adapt to dynamic stress environments under CSA systems. The fungal examples discussed in this section represent those for which hormonal interactions have been most clearly described in the literature. While not exhaustive, they capture the principal mechanisms through which endophytes regulate plant hormones under abiotic stress. Additional examples of endophytic fungi known to modulate key plant hormones are summarised in Table 1.

**Table 1.** Representative endophytic fungi reported to influence major plant hormones across different hosts and stress contexts. Examples illustrate the diversity of hormone-modulating mechanisms relevant to plant stress adaptation within climate-smart agriculture frameworks.

Fungal species	Key hormones	Host plants	Reported physiological effect	References
*Cyanodermella asteris*	IAA	*Arabidopsis thaliana*	Enhanced root elongation and biomass	[35]
*Trichoderma asperellum*	ACC deaminase, ET reduction	*Triticum aestivum*	Delayed senescence and improved waterlogging tolerance	[34]
*Aspergillus flavus,* *A. japonicus*	IAA, GA	*Glycine max,* *Helianthus annuus*	Growth promotion under heat stress	[30]
*Piriformospora indica*	ABA, SA	*Arabidopsis thaliana,* *Zea mays*	Improved drought/salt tolerance	[22,36]
*Fusarium solani*	IAA, JA	*Thymus vulgaris*	Increased antioxidant enzyme activity	[27]
				

### 2.5. Molecular and epigenetic mechanisms

**Figure 1.**
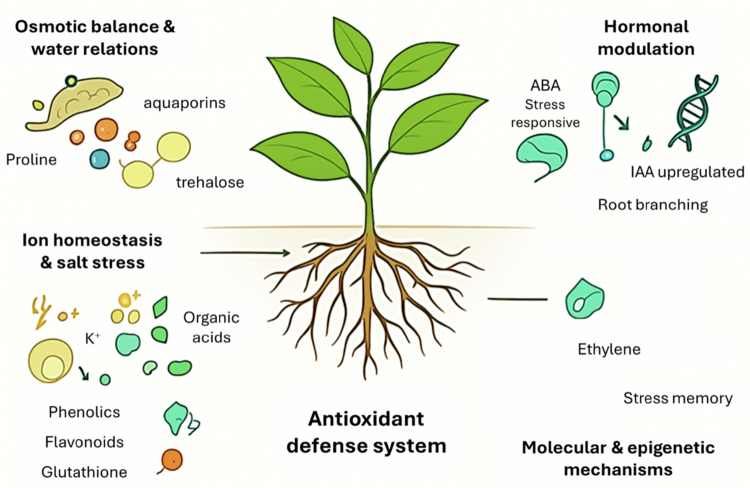
Mechanistic basis of endophytic fungal mediation in plant abiotic stress resilience. Endophytes enhance drought, salinity, oxidative, and temperature stress tolerance through multiple pathways, including osmotic regulation, ion homoeostasis, antioxidant defence, hormonal modulation, and molecular/epigenetic mechanisms. Figure created by Ramatsitsi *N* using Canva and Microsoft PowerPoint, adapted from conceptual frameworks in Vahabi et al.^22^, Rauf et al.^34^, and Zhang et al.^36^

Beyond physiological and biochemical pathways, endophytic fungi influence plant responses at the molecular and epigenetic levels. Transcriptomic studies reveal that endophytes induce the expression of stress-responsive genes involved in osmolyte biosynthesis, ROS detoxification, and ion transport.^37^ For example, *P*. *indica* upregulated heat and drought-inducible genes in maize, enhancing tolerance under water scarcity.^36^ Fungal endophytes also influence plant microRNA activity, altering gene silencing pathways associated with stress adaptation.^38^ According to research by Hubbard et al.^39^ colonisation of the endophytic fungus SMCD 2206 was associated with alterations in DNA methylation in drought-stressed wheat in the endosymbiotic seed-fungus connection. As shown across diverse hosts, from model plants such as *A. thaliana* to staple crops like *O. sativa*, *T. aestivum*, and *G. max*, the physiological and molecular outcomes of endophyte interaction are strongly host-dependent, a key consideration for translating laboratory findings into CSA field applications. Such epigenetic modifications such as DNA methylation and histone acetylation, triggered by fungal colonisation, can create a “stress memory” in plants, enabling faster and stronger responses upon recurrent stress exposure.^40^ These molecular interactions provide a longer-term adaptive advantage, extending beyond immediate physiological benefits. Importantly, such mechanisms position endophytes as potential bioengineering tools for developing resilient crop varieties within CSA frameworks. However, these processes are less understood compared to physiological pathways, highlighting the need for more omics-driven mechanistic studies. Endophytic fungi mediate plant adaptation through multiple mechanisms spanning osmotic regulation, ion homoeostasis, antioxidant defence, hormonal signalling, and epigenetic reprogramming (Figure 1). These hormonal and molecular interactions collectively enhance plant water-use efficiency, nutrient acquisition, and stress recovery, key outcomes aligned with CSA’s goals of improving productivity and adaptation while reducing chemical input requirements (i.e., mitigation). By linking molecular mechanisms such as hormone modulation and gene activation to field-level resilience traits, endophytic fungi can thus serve as practical biological components of CSA systems.

## 3. Comparative roles of major fungal lineages in stress regulation

Fungi are classified into seven major phyla: Ascomycota, Basidiomycota, Zygomycota, Chytridiomycota, Glomeromycota, Blastocladiomycota, and Neocallimastigomycota.^41^ While endophytic fungi are distributed across multiple phyla, Ascomycota and Basidiomycota are the most prevalent and ecologically influential in plant–fungus associations, with Zygomycota representing a smaller, yet notable component. Studies have shown that nearly 90% of endophytic fungi belong to Ascomycota, while Basidiomycota and Zygomycota are less frequently encountered in endophytic communities. The review is limited to these phyla given their predominant occurrence and central ecological significance in plant–fungus symbioses. The following sections summarise representative fungal endophytes within major taxonomic groups (Ascomycota, Basidiomycota, and Zygomycota) and their experimentally validated roles in abiotic stress mitigation. Where available, examples include species that have been evaluated under field or semi-field conditions, such as *Trichoderma harzianum*, *T. asperellum*, and *Serendipita indica* (previously known as *Piriformospora indica*). Other taxa discussed remain primarily studied under controlled environments, highlighting their potential but not yet confirmed applicability in field-based CSA frameworks.

### 3.1. Ascomycota

The Ascomycota represent the most diverse and abundant fungal lineage among plant endophytes, with numerous genera playing key roles in stress tolerance. Their primary strength lies in the production of a wide range of bioactive metabolites, including antioxidants, osmolytes, and phytohormones, which collectively improve host performance. Ascomycetes such as *A. aculeatus, Fusarium* spp. [unspecified] and *T. harzianum* often upregulate antioxidant enzymes such as superoxide dismutase and catalase, limiting ROS accumulation during drought or salinity stress.^12^^,^^17^^,^^27^ Hormonal modulation is another hallmark, with IAA synthesis promoting root development and ABA regulation enhancing stomatal control.^30^ These fungi are also metabolically flexible, adapting to diverse host plants and soil environments. Members of *Fusarium*, such as *F. solani* and *F. oxysporum*, have been reported to improve antioxidant activity, enhance nutrient acquisition, and promote host growth under drought and salinity stress.^27^^,^^29^ However, it is important to note that these benefits are strain-specific: only non-pathogenic or endophytic isolates exhibit such mutualistic behaviour. Accordingly, some taxa including those within the *Fusarium* group,^42^ complicate their direct application in agriculture. Despite this challenge, the Ascomycota remain central to mechanistic studies of stress mitigation due to their versatility and experimental tractability.

### 3.2. Basidiomycota

Basidiomycota endophytes, although less abundant than Ascomycota in many crops, exhibit specialised mechanisms that strongly support stress resilience. Notably, *S. indica* is well-characterised for their ability to enhance salinity and drought tolerance in cereals and legumes.^22^^,^^36^ These fungi often act by restructuring plant cell walls and producing extracellular polysaccharides that improve water retention and ion regulation. Their enzymatic capacity for lignin degradation also contributes indirectly to stress tolerance, as it facilitates nutrient recycling and root colonisation. Basidiomycetes are especially effective in root–fungus signalling, often leading to increased root biomass and improved resource acquisition under stress.^13^ Despite these strengths, their practical application in agriculture faces challenges: they are harder to culture, slower-growing, and less represented in comparative omics studies compared to Ascomycota. Nonetheless, their symbiotic efficiency and unique biochemical pathways make them valuable candidates for CSA-oriented bioinoculant development.

### 3.3. Zygomycota

Zygomycota, now largely reorganised under Mucoromycota, remain understudied as endophytes but show intriguing potential in abiotic stress mitigation. Multiple species within the genera *Penicillium,*^43^ and *Mortierella alpina*^44^ are fast-growing fungi capable of phosphate solubilisation, ion chelation, and organic acid secretion, which improve plant nutrient status under stress. Their rapid colonisation allows plants to establish early protective interactions, particularly under drought or nutrient-limited conditions. Unlike Ascomycota and Basidiomycota, however, the mechanistic understanding of their role in oxidative stress regulation, hormonal modulation, and epigenetic control is limited. Most evidence arises from laboratory studies, with relatively few field validations in agricultural systems. Given their capacity for nutrient mobilisation and resilience under stress, further exploration of Zygomycota could open new avenues for CSA practices, particularly in low-input farming systems where nutrient acquisition and water stress are major limitations. Among the fungal genera discussed, *T. harzianum* and *S. indica* have demonstrated reproducible performance under greenhouse and limited field trials, whereas others such as *Aspergillus* and *Fusarium* endophytes remain at the experimental stage. Continued evaluation of their ecological fitness and biosafety is essential for advancing their integration into climate-smart agricultural systems.

### 3.4. Comparative insights

**Figure 2.**
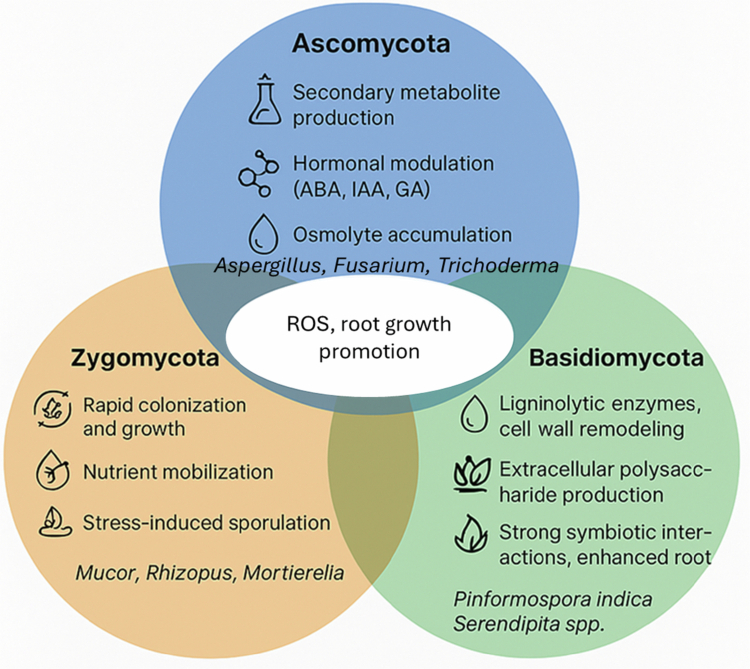
Comparative roles of major fungal lineages in abiotic stress regulation. Ascomycota, Basidiomycota, and Zygomycota differ in their dominant mechanisms, yet converge in promoting plant growth, abiotic stress resilience, and CSA potential.

Collectively, Ascomycota, Basidiomycota, and Zygomycota contribute to abiotic stress regulation in distinct but complementary ways. Ascomycota dominate through metabolic versatility and secondary metabolite production, Basidiomycota specialise in symbiotic efficiency and cell wall interactions, and Zygomycota offer rapid growth and nutrient mobilisation potential. These three phyla exhibit both unique and overlapping mechanisms in stress regulation, with distinct associations to specific abiotic stressors (Figure 2, Table 2).

**Table 2.** Plant stress regulation mechanisms facilitated by Ascomycota, Basidiomycota, and Zygomycota phyla.

Fungal lineage	Dominant traits in stress regulation	Key mechanisms	Representative genera	Stress types addressed	Limitations/gaps	Application status/validation level
Ascomycota	Highly diverse, dominant in agricultural endophytes	Secondary metabolite production, ROS scavenging, hormonal modulation (e.g., IAA, ABA), osmolyte accumulation	*Trichoderma, Fusarium, Aspergillus*	Drought, salinity, heavy metals	Mechanistic studies uneven across taxa; some pathogenic spp. complicate application	*Trichoderma* spp. extensively validated in greenhouse and field conditions; several commercial formulations available. Non-pathogenic *Fusarium* and *Talaromyces* isolates tested experimentally; *Aspergillus* mostly studied under controlled conditions.
Basidiomycota	Symbiotic associations	Ligninolytic enzymes, cell wall remodelling, enhanced root growth, polysaccharide production,	*Piriformospora indica*, *Serendipita* spp., *Rhizoctonia* spp.	Salinity, heat, drought	Fewer crop-focused studies; harder to culture *in vitro* compared to Ascomycota	*Serendipita indica* validated in multiple greenhouse and semi-field trials with cereals and legumes; promising candidate for CSA applications though field data remain limited.
Zygomycota	Rapid growth, nutrient mobilisation	Stress-induced sporulation, ion chelation, phosphate solubilisation	*Mucor*, *Rhizopus*, *Mortierella alpina*	Drought, nutrient stress, some heavy metals	Understudied in endophytic context; mechanisms less characterised; mostly lab evidence	Primarily lab-scale investigations; early studies indicate potential in soil-amendment or biofertiliser formulations, but no field-level validation yet.

ROS = reactive oxygen system; IAA = indole acetic acid; ABA = abscisic acid.

## 4. Integrating endophytic fungi into CSA practices

The CSA system encompasses three interlinked objectives; productivity, adaptation, and mitigation, that can be achieved through a combination of agronomic and biological strategies. Key practices include crop diversification, residue retention, reduced tillage, and organic-matter enrichment, all of which influence soil microbial communities. Endophytic fungi contribute to these strategies by improving nutrient-use efficiency, enhancing stress resilience, and reducing chemical input dependency. For instance, *T. asperellum* and *T. harzianum* formulations have been successfully integrated into conservation agriculture systems to enhance root growth and carbon stability, while *S. indica* supports drought-adapted cereal production under minimal irrigation.^22^^,^^36^

The incorporation of endophytic fungi into CSA offers a promising biological strategy to enhance crop resilience while reducing environmental footprints. As demonstrated by Sorahinobar et al.,^12^ *T. harzianum* regulated *H. vulgare* root aquaporin expression, thereby improving water uptake and sustaining photosynthesis under drought stress. Likewise, argued that fungal-derived IAA drives root branching and elongation, increasing soil exploration and nutrient acquisition. These findings illustrate how Ascomycota lineages, with their metabolic versatility, underpin CSA’s adaptation pillar through osmotic regulation and hormonal modulation. Basidiomycota endophytes, though less diverse, often function as symbiotic specialists. For example, *P. indica* promotes sodium sequestration into vacuoles and modulates stress-inducible gene expression in cereals, enhancing tolerance to salinity and drought.^13^ Such dual physiological and molecular effects position Basidiomycota as valuable partners for crop resilience in stress-prone agroecosystems. Endophytes also advance CSA’s mitigation goals. Singh et al.^45^ indicated that fungal metabolites contribute to soil organic matter stabilisation, while Sun et al.^46^ reported that *F. oxysporum* endophytes enhance antioxidant enzyme activity, lowering oxidative crop losses during heat stress. In parallel, Zygomycota species such as *M. alpina* improve phosphate solubilisation and nutrient cycling, a function that Wang et al.^47^ argued is especially relevant for smallholder systems reliant on low external inputs.

Comparatively, Ascomycota strains often deliver faster colonisation and higher biochemical turnover, whereas Basidiomycota provide longer-term symbiotic stability and drought endurance, highlighting the need for context-specific selection rather than a one-size-fits-all approach. Collectively, these findings highlight how endophytes reduce dependency on synthetic agrochemical, improve soil health, and lower the carbon intensity of farming systems. From a CSA perspective, this diversity suggests that multi-lineage consortia may provide more robust resilience than single-strain inoculants, as they could simultaneously target multiple stress pathways. However, uneven research focus, heavily favouring Ascomycota, limits our ability to fully exploit the functional breadth of these lineages. Future CSA strategies should integrate lineage-specific strengths while addressing gaps, especially in the underexplored Basidiomycota and Zygomycota. The use of microbial consortia, combinations of fungi, bacteria, or both represents an emerging CSA strategy that leverages synergistic interactions. Co-inoculation of *Trichoderma harzianum* with *Bacillus subtilis* or *Rhizobium* spp. has shown improved plant water status and nutrient uptake under drought conditions.^40^ Similarly, *S. indica* combined with arbuscular mycorrhizal fungi (*Rhizophagus irregularis*) enhances phosphorus acquisition and yield stability in cereals. *Mortierella alpina* has been trialled with *Azotobacter chroococcum* for simultaneous nitrogen fixation and phosphate solubilisation in legumes. Such combinations demonstrate high potential for integrated CSA systems, though field-scale validation and formulation stability remain key challenges.

Despite these opportunities, practical application faces hurdles. Christian et al.^48^ cautioned that host–fungus specificity and environmental variability often lead to inconsistent outcomes in field settings. Qaderi & Safaie^25^ and de Lamo & Takken^49^ further emphasised that the limited stability of inoculant formulations and the dual pathogenic potential of some taxa, such as *Fusarium*, complicate their large-scale use. Addressing these limitations will require advances in omics-enabled strain discovery, next-generation inoculant technologies, and multi-location trials to validate effectiveness under diverse agroecological conditions. Farmer adoption depends on affordability of bioinoculant products, knowledge-sharing through extension services, and incentives to reduce reliance on chemical inputs. Regulatory clarity surrounding microbial inoculants, alongside investment in public–private partnerships, will be essential for scaling production and distribution. As Patel et al.^40^ noted in the context of plant stress memory, long-term resilience requires not only biological innovation but also institutional continuity. Incorporating endophytic fungi within CSA practices therefore represents more than a technical solution, it reflects a systems approach to building resilient, adaptive, and low-carbon food production in the face of climate change. The alignment of endophytic functions with CSA strategies highlights a promising pathway for scalable biological solutions.

## 5. Applied prospects and challenges for bioinoculant development

The growing evidence for the role of endophytic fungi in plant stress resilience has stimulated interest in their application as bioinoculants for agriculture. Unlike synthetic agrochemicals, fungal bioinoculants represent living tools that can establish symbiotic relationships, persist in plant tissues, and respond dynamically to environmental conditions. Their potential to provide protective measures against abiotic stress makes them especially attractive for CSA systems. However, the pathway from laboratory discovery to widespread agricultural deployment remains complex, with several opportunities and challenges shaping their applied prospects. The preceding chapter outlined CSA practices such as conservation tillage, organic amendments, and crop diversification. These strategies provide the operational context in which endophytic fungi can be deployed as biological tools for stress adaptation and input reduction.

***Formulation and delivery systems:*** The practical success of endophyte-based inoculants depends on robust formulation and delivery technologies. Unlike bacterial bio-fertilisers, several endophytic fungi, including *Cladosporium oxysporum* and *Rhizoctonia solani* have slower growth rates, complex nutritional requirements, or limited stability outside their host.^24^ Formulation strategies such as seed coatings, encapsulation, or incorporation into carrier substrates (peat, biochar, or alginate beads) are being explored to improve shelf-life and ease of application.^25^^,^^50^ Encapsulation is particularly promising, as it not only preserves viability but also enables controlled release of fungal propagules under field conditions.^51^ Comparatively, seed coating remains the most scalable and cost-efficient option for cereals and legumes, while encapsulation offers superior microbial protection but higher production costs. Biochar-based carriers show intermediate stability with added soil-health benefits, though field performance data are still limited. Advances in nanotechnology^52^ and polymer chemistry^53^ may further improve delivery efficiency, though these approaches are still in their infancy with regards to crop resilience applications.

***Compatibility with crops and microbial consortia:*** An additional consideration is compatibility across crop species. Wu et al.^26^ contended, several endophytic effects are host-genotype specific, suggesting that a strain highly effective in one crop may show negligible performance in another. This restricts the development of universal products. A potential solution lies in designing microbial consortia that combine complementary fungal lineages; Ascomycota for metabolite diversity, Basidiomycota for symbiotic signalling, and Zygomycota for nutrient mobilisation. Such consortia could provide broader and more consistent benefits across diverse agroecosystems. However, co-culturing multiple fungi presents challenges in terms of stability, competition, and maintaining functional balance, requiring more research into community–level dynamics. In practice, mixed consortia often outperform single-strain inoculants under variable field conditions but pose higher risks of contamination and shelf-life reduction, highlighting a key trade-off between functional diversity and quality control.

***Comparison with other microbial inoculants:*** While mycorrhizal fungi and rhizobacteria have already gained traction in commercial inoculant markets, endophytic fungi remain comparatively underexplored.^54^ One primary advantage is their ability to colonise internal tissues rather than remain confined to the rhizosphere, offering more direct protection against stress.^8^ However, their dual identities complicate commercialisation: taxa such as *F. oxysporum* or *Aspergillus* spp. [unspecified species] include both beneficial and pathogenic strains^25^^,^^49^ necessitating careful strain selection and biosafety screening. Compared with arbuscular mycorrhizal fungi (AMF) and plant-growth-promoting rhizobacteria (PGPR), fungal endophytes offer broader colonisation niches and more metabolic flexibility. AMF primarily improve phosphorus uptake and soil aggregation, whereas PGPR such as Bacillus subtilis and Pseudomonas fluorescens enhance nitrogen fixation, phytohormone production, and induced systemic tolerance. Endophytic fungi, including *T. harzianum* and *S. indica*, can persist inside plant tissues and provide dual roles as biostimulants and bioprotectants, often maintaining activity under fluctuating climatic conditions. Field comparisons indicate that co-inoculation of *Trichoderma* with AMF or Bacillus species can outperform single inoculants, improving drought and salinity tolerance as well as yield stability.^40^ While AMF are often slower to establish and sensitive to soil disturbance, endophytes are easily cultured and formulated, giving them a practical advantage for rapid production and deployment. However, ecological consistency remains a challenge; field responses of endophytes can vary more strongly with host genotype and environment than those of bacterial inoculants. Learning from the regulatory and market experiences of mycorrhizal and bacterial inoculants may accelerate the pathway for endophytes, but specific guidelines tailored to their biology are still lacking.

***Regulatory and adoption challenges:*** Institutional and regulatory frameworks represent another critical bottleneck. In many regions, microbial inoculants are regulated under fertiliser or pesticide laws^55^^,^^56^ that do not account for the unique biology of endophytes. This lack of clarity slows product approval and discourages investment. Farmer adoption also depends on awareness, access to affordable products, and trust in their efficacy. O'Callaghan et al.^57^ noted that without effective extension services, microbial solutions risk remaining confined to research plots. Public–private partnerships, supported by clear regulatory pathways, will be essential to bridge this gap and enable scaling. Clear biosafety standards, defining permissible strains, contamination thresholds, and environmental-release criteria, are also crucial for ensuring public confidence and international trade acceptance.

***Future applied prospects:*** Despite these challenges, the applied potential of endophytic fungi is considerable. Advances in omics and synthetic biology may allow the development of engineered strains with enhanced stress-mitigation capacities. Integration with precision agriculture technologies could further optimise inoculant use by tailoring applications to specific soils, climates, and crops. Importantly, the applied deployment of endophytes must be guided not only by mechanistic insights (Figures 1–2) but also by ecological compatibility and socio-economic feasibility. If these dimensions are addressed, fungal endophytes could move from experimental curiosities to integral components of CSA systems, supporting resilient, sustainable, and low-input farming worldwide. While strain engineering and synthetic biology approaches could enhance metabolite profiles or stress-protective functions, these tools raise legitimate biosafety and regulatory concerns. Engineered microorganisms face stricter approval procedures and social acceptance barriers than naturally occurring isolates, particularly in open-field use. From a practical standpoint, affordability also determines adoption: commercial fungal inoculants currently range from roughly USD 5 to 25 per hectare, affordable mainly for high-value crops. Local production and cooperative distribution systems may lower costs and make bioinoculants more accessible to smallholder farmers. Collectively, these considerations underscore the need for integrated evaluation frameworks that jointly assess formulation efficiency, field performance, biosafety, and scalability before commercial release. The major challenges and potential solutions for developing fungal bioinoculants are summarised in Table 3.

**Table 3.** Challenges and potential solutions in the development of fungal bioinoculants for CSA.

Challenge	Implication for CSA adoption	Potential solutions/strategies	Supporting references
Limited shelf-life and stability of formulations	Reduced viability during storage and transport	Encapsulation, seed coatings, polymer-based carriers, nanotechnology for controlled release	[50,51,53]
Host–genotype specificity of endophyte effects	Inconsistent performance across crops and regions	Omics-guided strain selections, development of crop-specific inoculants, multi-strain consortia design	[25, 26]
Coexistence of beneficial and pathogenic strains (e.g., *Fusarium*, *Aspergillus*)	Biosafety risks and reduced farmer trust	Rigorous screening pipelines, genome-based strain authentication, regulatory standards for safe strains	[40,52]
Challenges in culturing slow-growing lineages (e.g., Basidiomycota)	Limits commercial scalability and diversity of products	Improved culturing methods, synthetic media development, cryopreservation for long-term storage	[55,56]
Lack of tailored regulatory frameworks	Slows product approval and private-sector investment	Clearer policies for microbial inoculants, harmonised international guidelines, evidence-based biosafety assessments	[8,24,25]
Low farmer awareness and adoption barriers	Limited market demand and poor scaling	Extension programs, participatory field trials, subsidies/incentives for microbial inputs, public–private partnerships	[55‐57]
Low farmer awareness and adoption barriers	Limited market demand and poor scaling	Extension programs, participatory field trials, subsidies/incentives for microbial inputs, public–private partnerships	[9,57,58]

## 6. Knowledge gaps and future directions

Despite the fact that endophytic fungi have indispensable roles in mediating abiotic stress resilience is increasingly recognised, substantial gaps remain in translating mechanistic insights into agricultural practice. Several current studies rely on controlled laboratory or greenhouse conditions, where endophyte–host interactions are often more predictable than in heterogeneous field environments. As indicated by Chen et al.,^59^ the ecological complexity of natural soils, combined with variable climate pressures, makes field-level consistency a major challenge. Accordingly, large-scale field validation emerges as the most urgent research priority, requiring coordinated, multi-season and multi-location experiments across contrasting agroecological zones. Long-term, multi-location trials are therefore essential to validate strain performance under diverse agroecosystems and management practices. Another key gap lies in host specificity and context dependency. Other endophytic fungal effects have been shown to be plant genotype–dependent, with strains conferring strong benefits in one host but negligible or even detrimental effects in another. This dual lifestyles of certain taxa, such as *Fusarium* and *Aspergillus*^49^^,^​​​​​​^60^ further complicate application, as beneficial strains coexist alongside pathogenic relatives. Another priority is integrating multi-omics tools—genomic, transcriptomic, metabolomic, and epigenomic, to build predictive frameworks for safe strain selection and functional validation. Developing robust screening pipelines that integrate genomic, transcriptomic, and metabolomic data will be critical to identify safe and effective candidates for bioinoculant development.

At the mechanistic level, future research should move beyond physiological assays to dissect molecular and epigenetic processes. As demonstrated by Zhang et al.,^36^ *P*. *indica* can activate drought-inducible genes, but the breadth of transcriptional and small RNA-mediated regulation remains poorly understood. Similarly, the epigenetic “stress memory” effects reported by Hoenicka et al.^61^ have yet to be systematically explored in fungal–plant systems. Leveraging CRISPR-based tools and single-cell sequencing could provide unprecedented insights into how endophytes reprogramme plant stress responses at fine resolution (Figure 1). Most endophytes studied remain uncultured or difficult to stabilise in commercial products, particularly Basidiomycota lineages with slow growth rates. Encapsulation technologies, synthetic microbial consortia, and seed-coating approaches may offer scalable solutions, but these remain underexplored compared to bacterial inoculants. Scalability therefore represents a third major frontier, demanding innovation that links biological optimisation with low-cost formulation and local production capacity. Future innovations should also consider lineage-specific traits outlined in Figure 2, ensuring that bioinoculant design matches fungal ecological function.

Furthermore, integrating endophytes into CSA frameworks will require interdisciplinary approaches that bridge biology, agronomy, and policy. Cross-disciplinary collaboration among molecular biologists, agronomists, economists, and policymakers is essential to translate mechanistic discoveries into deployable solutions. As other authors have contended,^9^^,^^58^, fungal endophytes align strongly with CSA’s pillars, yet institutional barriers, ranging from regulatory uncertainty to limited extension services, continue to slow adoption. Creating shared data platforms, farmer-oriented demonstration networks, and regional regulatory dialogues could further align research outcomes with policy and practice. Establishing farmer-oriented research platforms and fostering public–private partnerships could accelerate the translation of promising strains into field-ready technologies. Overall, prioritising field validation, omics-driven discovery, and cross-sector collaboration provides a strategic roadmap for advancing endophytic fungi from experimental systems to climate-smart agricultural practice. Future research priorities are summarised in Table 4, showing how each aligns with CSA’s triple pillars.

**Table 4.** Future research priorities for integrating endophytic fungi into CSA systems.

Research priority	Key rationale	Link to CSA pillars
Multi-location and long-term field trials	Ensure consistency across climates and soils	Productivity, adaptation
Omics-driven strain screening	Identify safe, effective, non-pathogenic candidates	Productivity, adaptation
Molecular and epigenetic mechanisms	Clarify stress memory, small RNA regulation	Adaptation
Advanced formulations (encapsulation, seed-coating, synthetic consortia)	Improve shelf-life, delivery, and scalability	Productivity, mitigation
Policy and extension frameworks	Promote adoption through incentives and partnerships	Productivity, adaptation, mitigation

## 7. Concluding remarks

Endophytic fungi represent a vital yet underutilized resource for enhancing plant resilience to abiotic stresses in the face of climate change. As this review has demonstrated, they modulate plant physiology through diverse mechanisms, including osmotic regulation, ion homoeostasis, antioxidant defence, hormonal modulation, and molecular reprogramming. These processes not only sustain photosynthesis, growth, and survival under drought, salinity, heat, and heavy metal stress but also align directly with the three pillars of CSA, i.e., productivity, adaptation, and mitigation. However, the transition from laboratory insights to field-scale applications remains largely unrealised. Variability in host–endophyte compatibility, the endophyte–pathogen continuum observed in certain fungal taxa, and technical barriers to inoculant formulation constrain their widespread adoption. Advancing this field will require integrating omics-driven research with practical agronomic evaluations, alongside supportive policies and farmer-oriented innovation platforms. Future perspectives suggest that endophytic fungi offer more than short-term stress alleviation; they provide opportunities for adaptive capacity and ecological resilience within agricultural systems. To translate mechanistic understanding into practice, future work should prioritise (i) multi-site validation of key fungal strains across representative crops, (ii) development of standardised screening and formulation protocols, and (iii) creation of regional bioinoculant hubs to support local production and training. Strengthened collaboration among researchers, policymakers, and extension networks will be critical for mainstreaming endophyte-based interventions into CSA frameworks. Through harnessing their symbiotic potential, CSA can move beyond incremental efficiency gains toward a deeper transformation of crop management under climate uncertainty. Thus, endophytic fungi should be regarded not merely as experimental curiosity but as cornerstone allies in the quest for sustainable and resilient food systems.

## Acknowledgments

Not applicable.

## Data Availability

Data sharing not applicable to this article as no datasets were generated or analysed during the current study.
